# Critical care issues in liver transplantation

**DOI:** 10.4103/0972-5229.68232

**Published:** 2010

**Authors:** Luis Ignacio Gonzalez-Granado

**Affiliations:** Immunodeficiencies Unit, Hospital 12 octubre. Carretera Andalucia km 5,400. Postal Code: 28041. Madrid, Spain

Dear Editor,

I read with keen interest the review by Gopal *et al*. in the journal.[1] I am grateful for their great review. However, I would like to make some comments.

First, there are recent reports that demonstrate the superiority of quadruple or triple immunosupression versus traditional approach in terms of efficacy and safety.[2] This positive outcome is mainly related to the use of tacrolimus as the cornerstone within the immunosuppressive treatment.[3]

Second, in areas where Chagas disease is endemic, with migratory flows, reactivation should be considered when the donor has Latin-American origin, nowadays this complication has been recognized in USA and Europe.[4–5]

Third, the authors missed the important issue that transplant recipients may develop severe infection with Streptococcus pneumoniae, even in the early postransplant period.[6] Vaccination has been recommended in heart, renal and liver recipients. Available vaccines are the 23-valent polysaccharide and the hepta- and decavalent protein conjugate.[7]
